# Supplementary material for: Comparison of the clinical value of MRI and plasma markers for cognitive impairment in patients aged ≥75 years: a retrospective study
Source: PeerJ. 2023 Jun 22;11:e15581. doi: 10.7717/peerj.15581 (PMC10290829; doi:10.7717/peerj.15581)
Supplement: Supplemental Information 2 [file peerj-11-15581-s002.docx]

**Supplementary Table S1.** MRI scanning protocol in different MRI scanners.

| Scanner | Axial T1WI | Axial T2WI | Axial T2 FLAIR | Axial DWI |
| --- | --- | --- | --- | --- |
| GE MEDICAL SYSTEMS  Discovery MR 750w 3.0T | TR=2000 ms  TE=24.96 ms  FOV=240 mm  matrix=224×320  ST=6 mm | TR=4356 ms  TE=103 ms  FOV=240 mm  matrix=416×416  ST=6 mm | TR=6500 ms  TE=103 ms  FOV=240 mm  matrix=256×256  ST=6 mm | TR=3641 ms  TE=78 ms  FOV=240 mm  matrix=130×169  ST=6 mm |
| GE MEDICAL SYSTEMS  Signa HDxt 3.0T | TR=2136 ms  TE=22 ms  FOV=240 mm  matrix=224×320  ST=6 mm | TR=4000 ms  TE=94 ms  FOV=240 mm  matrix=384×384  ST=6 mm | TR=8000 ms  TE=168 ms  FOV=240 mm  matrix=224×288  ST=6 mm | TR=5000 ms  TE=74 ms  FOV=240 mm  matrix=160×160  ST=6 mm |
| Philips MEDICAL SYSTEMS Ingenia 3.0T | TR=2000 ms  TE=20 ms  FOV=230 mm  matrix=196×168  ST=6 mm | TR=2123 ms  TE=80 ms  FOV=230 mm  matrix=266×320  ST=6 mm | TR=9000 ms  TE=110 ms  FOV=230 mm  matrix=193×208  ST=6 mm | TR=2773 ms  TE=103 ms  FOV=230 mm  matrix=122×152  ST=6 mm |
| Philips Medical systems Achieva 1.5T | TR=2000 ms  TE=24 ms  FOV=230 mm  matrix=205×256  ST=6 mm | TR=4280 ms  TE=120 ms  FOV=230 mm  matrix=235×288  ST=6 mm | TR=6000 ms  TE=120 ms  FOV=230 mm  matrix=198×256  ST=6 mm | TR=2706 ms  TE=94 ms  FOV=230 mm  matrix=129×144  ST=6 mm |
| TOSHIBA-MEC 1.5T | TR=2150 ms  TE=15 ms  FOV=240 mm  matrix=224×272  ST=6 mm | TR=3200 ms  TE=90 ms  FOV=240 mm  matrix=224×320  ST=6 mm | TR=8000 ms  TE=100 ms  FOV=240 mm  matrix=192×320  ST=6 mm | TR=4000 ms  TE=100 ms  FOV=240 mm  matrix=120×122  ST=6 mm |

TE, echo time; TR, repetition time; FOV, field of view; ST, slice thickness.

**Supplementary Table S2.** Summary of brain atrophy ^43^.

|  | Normal=0 | Mild=1 | Moderate=2 | Severe=3 |
| --- | --- | --- | --- | --- |
| Central atrophy (LVBI) | >4 | 3.6-4 | 3-3.5 | <3 |
| Cortical atrophy (sulci width) |  | 3-5 | >5 | >6 |

**Supplementary Table S3.** The ICC of linear indicators for central brain atrophy

| Variables | ICC | P value |
| --- | --- | --- |
| a | 1 | **<0.001** |
| b | 0.996 | **<0.001** |

a: minimum distance of lateral wall of bilateral lateral ventricle; b: brain transverse diameter at the same level; ICC: interclass correlation coefficient; bold means P < 0.05.

**Supplementary Table S4.** Results of ROC analysis for multiple indicators combined diagnosis in different group.

| Group Variables | Cut off | AUC | 95%CI | SE (%) | [SP](javascript:;) (%) |
| --- | --- | --- | --- | --- | --- |
| PVWMH, LVBI, and cortical atrophy（mode 1） | 0.342 | 0.782 | 0.716-0.840 | 68.5 | 78.5 |
| PVWMH and LVBI(mode 2) | 0.341 | 0.734 | 0.640-0.815 | 71.0 | 77.6 |
| PVWMH, LVBI, and cortical atrophy(mode 3) | 0.259 | 0.757 | 0.674-0.828 | 83.8 | 65.6 |
| PVWMH and LVBI(mode 4) | 0.360 | 0.720 | 0.618-0.808 | 69.0 | 76.9 |

mode 1: CN vs cognitive impairment; mode 2: Aβ- subgroup; mode3: tau- subgroup; mode 4: Aβ-tau- subgroup; AUC: area under ROC curve; 95%CI: 95% Confidence interval; SE: sensitivity; SP: specificity.

**Figure Legends**

**Supplementary Figure S1.** Representations of grades 0, 1, 2, and 3 for DWMH and PVWMH.
