# Supplementary figures and images for: Comparison of the clinical value of MRI and plasma markers for cognitive impairment in patients aged ≥75 years: a retrospective study
Source: PeerJ. 2023 Jun 22;11:e15581. doi: 10.7717/peerj.15581 (PMC10290829; doi:10.7717/peerj.15581)

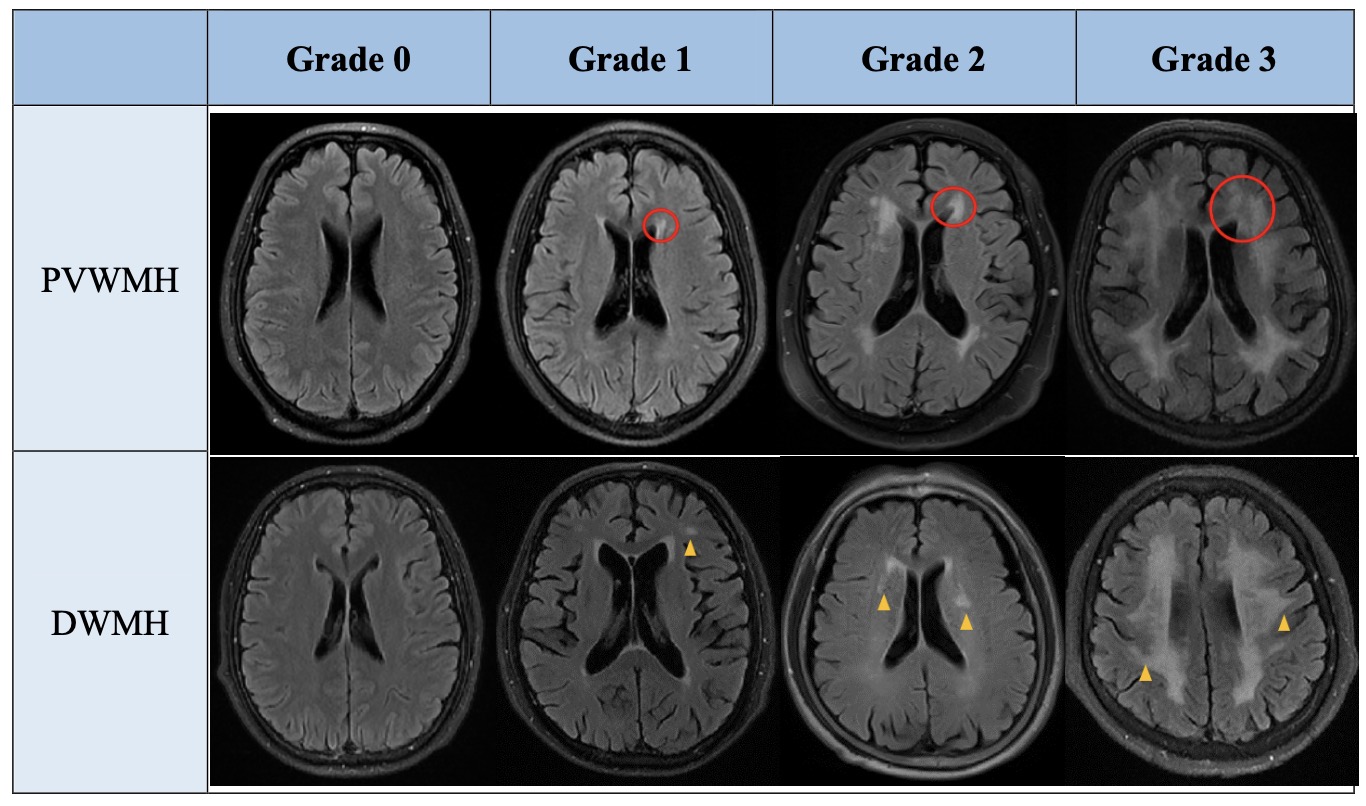

Supplement: Supplemental Information 3 [file peerj-11-15581-s003.jpg]
