# Supplementary material for: Comparison of the clinical value of MRI and plasma markers for cognitive impairment in patients aged ≥75 years: a retrospective study
Source: PeerJ. 2023 Jun 22;11:e15581. doi: 10.7717/peerj.15581 (PMC10290829; doi:10.7717/peerj.15581)
Supplement: Supplemental Information 4 — Patients with a clinical diagnosis of CI (n = 54) (yellow) and without CI (n = 130) (blue) were enrolled, clinical variables including plasma indicators (Aβ42 and p-tau181) and conventional structural MRI parameters were collected and analyzed. We found that the CI group (yellow) had higher WMH load, smaller LVBI value, and more severe cortical atrophy compared to the non-CI (NCI) group (blue), while plasma Aβ42 and p-tau181 showed no significant difference between the two groups. The combined model including PVWMH, LVBI, and cortical atrophy to detect CI and NCI showed an area under the ROC curve is 0.782. [file peerj-11-15581-s004.pdf]

Dementia has become the main cause of disability in older adults aged ≥75 years. Cerebral small vessel disease (CSVD) is a cause of vascular cognitive impairment (VCI).

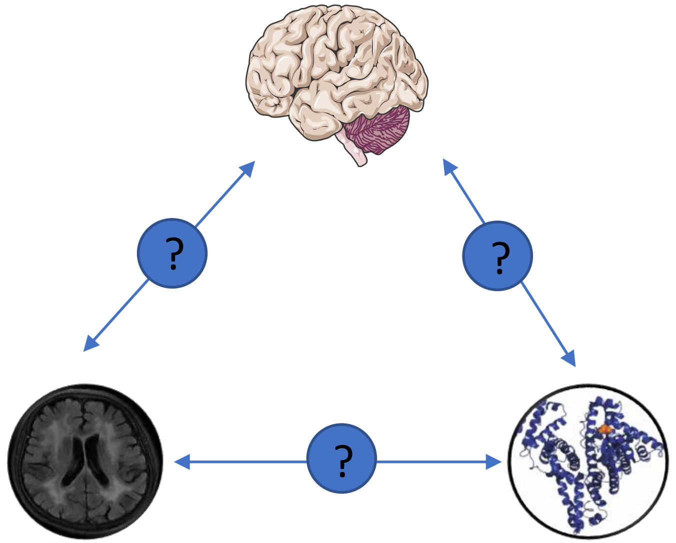

WMH and atrophy **plasma Aβ42 and P-tau 181**

Plasma Aβ42 and p-tau181 are associated to multiple Alzheimer's disease-related cognitive domains. However, their relationship in CSVD is still unknown.

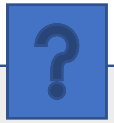

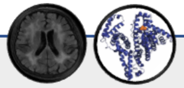

Cost-effective, non-invasive markers will be beneficial to clinical, scientific research and long-term follow-up. Do plasma Aβ42, p-tau181 and conventional MRI parameters have any clinical value in evaluating CI in patients aged ≥75 years?

Group 54 patients with CI 130 patients without CI

Measure

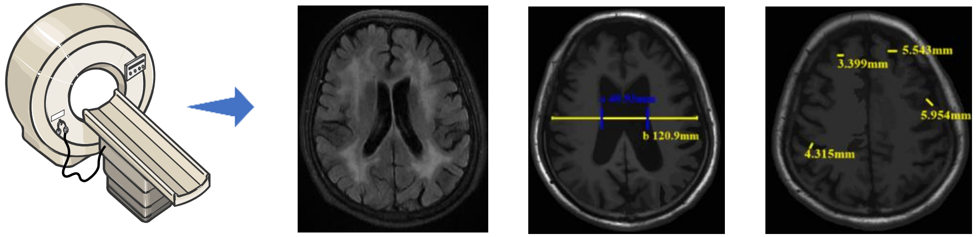

WMH **central atrophy** **cortical atrophy**

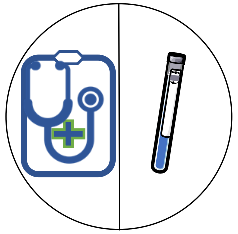

Clinical Variables including plasma Aβ42 and P-tau 181

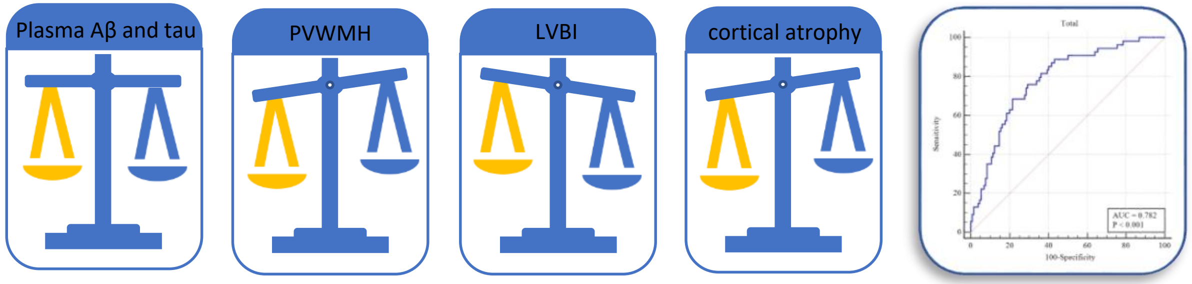

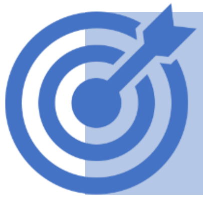

Conclusion: Plasma Aβ42 and P-tau181 might not be associated with CI, while MRI markers are related to CI.
